# Associations Among Attention Problems, Learning Strategies, and Hazardous Drinking Behavior in a College Student Sample: A Pilot Study

**DOI:** 10.1177/1178221819848356

**Published:** 2019-05-15

**Authors:** Jennifer Bolden

**Affiliations:** Department of Psychology, University of Tennessee, Knoxville, TN, USA

**Keywords:** hazardous drinking behavior, learning, attention problems

## Abstract

Despite research linking substance use/abuse to pejorative academic outcomes, the underlying behavior and cognitive mechanisms responsible for this association are largely unknown. This study addresses a specific call for understanding learning strategies and skills associated with substance alcohol use/abuse. Four hundred fifty undergraduates (59.6% female) completed measures of hazardous drinking behavior and student learning strategies. Approximately 35.3% of the sample reported hazardous drinking scores in the clinical range. Bivariate correlations and a regression framework were utilized to understand the associations among hazardous drinking behavior, academic skills/strategies, and student liabilities. In the present study, hazardous drinking behavior was associated with 4 learning strategies: note-taking/listening skills, test-taking strategies, organizational techniques, and time management. Moreover, hazardous drinking behavior was associated with 2 student liabilities: low academic motivation and concentration/attention difficulties. Results from follow-up analyses suggest that only organizational techniques and concentration/attention difficulties predicted hazardous drinking behavior. Promising areas for future research and potential intervention targets are discussed.

Alcohol is the most commonly used drug on college campuses, with over 60% of undergraduates consuming alcohol in the past month^1^ and approximately 43% of college students experiencing at least one heavy or binge drinking episode within the past 30 days.^2^ Alcohol abuse on college campuses is an emerging campus health concern^1^ and understanding alcohol use/abuse on college campuses is important. Extant research links alcohol consumption to numerous negative outcomes including both health-related and legal concerns. Approximately 10% of all emergency room visits at a large university medical center are alcohol related.^3^ Moreover, approximately 500 000 college students in the United States report alcohol-related injuries and alcohol is linked to more than 1700 deaths.^4^ Similarly, alcohol-related problems are associated with lower levels of general life satisfaction.^5^ Binge drinking is related to legal problems (eg, damaging property, driving under the influence) and alcohol use is linked to both social difficulties (eg, arguing with friends) and health impairments (eg, unplanned and/or unprotected sexual activity, lower immunity, and decreasing physical health).^6^

While extant research links problematic drinking behavior to health, legal, and social problems, it is important to understand the associations between alcohol use/abuse and academic-related concerns. McGee and Kypri^7^ documented an association between hazardous drinking behavior and four academic-related problems: completing assignments in a timely fashion, concentrating in class, missing class, and being late for class. Similarly, Presley and colleagues^8^ documented consistent near-term academic correlates of hazardous drinking behavior. They noted associations among alcohol consumption, performing poorly on a test/project, and missing a class during the academic year.^8^ In a report to college presidents, Presley and Meilman noted that long-term academic consequences of alcohol use/abuse include college retention problems, lower overall grade point averages (GPA), and academic probation.^9^ Moreover, a relationship between alcohol consumption and GPA is documented even after controlling for Scholastic Aptitude Test (SAT) scores and high school class rank.^10^

Although research links hazardous drinking behavior to near- (ie, missing class) and long-term academic consequences (ie, lower GPA, probation status), the underlying behavioral and cognitive mechanisms responsible for this association are largely unknown. Researchers posit that substance use impairs both learning and memory, particularly the development of higher-order cognitive processes (ie, executive functions) that are critical to learning and navigating the transition to adulthood. These higher-order processes include inhibition and planning/organization and are essential for problem-solving and guiding everyday behavior.^11^ Moreover, alcohol-related disorders are associated with visual spatial problems^12,13^ and concerns with psychomotor speed and coordination.^13^ Researchers posit that substance use/abuse is associated with impaired study habits or skills which may, in turn, lead to long-term academic consequences.^11^ While hazardous drinking behavior is associated with impairments in executive functions, no study, to date, has examined the associations between hazardous drinking behavior and specific study strategies.

It is important to note that while study strategies and study skills are often used interchangeably, study skills are global in scope and refer to a task-specific subset of behaviors that facilitate the learning (ie, storage and retrieval) of presented material.^14^ Learning strategies, however, are related to specific cognitive tactic for storing/encoding and processing stored information.^15^ Specific learning strategies include study strategies, note-taking/listening strategies, and reading comprehension strategies. Referred to as the “third pillar supporting collegiate academic performance,”^16^ study skills/learning strategies are informed by both indirect/distal determinants (eg, cognitive capacity, motivation/interests, and personality variables, and education/training experience) and direct/proximal determinants (eg, motivation and both declarative and procedural knowledge).^17^ Among adolescents, substance use/abuse is associated with attention problems and problems retrieving both verbal and visual material.^18^ While research documents positive outcomes for interventions aimed at improving learning strategies, most research on learning strategies has been conducted with adolescent students with identified learning disabilities.^14^ Despite concerns regarding the documented relation between hazardous drinking behavior and learning, the extent to which hazardous drinking behavior is related to specific study skills and learning strategies in a college student sample is unknown.

To expand existing knowledge, the present study attempts to understand the associations among hazardous drinking behavior, learning strategies, and student liabilities in a college student sample. Student liabilities are modifiable behaviors that affect the acquisition, processing, and retrieval of information.^14^ Academic-related student liabilities include test anxiety, attention problems, and poor student motivation. The present study addresses a specific call for understanding learning strategies and skills associated with hazardous drinking behavior^11^ and is the first study, to date, to examine the association between specific study skills/learning strategies and hazardous drinking behavior. The study aims to identify potential treatment targets and inform the development of impairment-specific academic interventions for college students at risk of engaging in hazardous drinking behavior. First, bivariate correlations were conducted to understand the associations among hazardous drinking behavior, school motivation, and learning strategies. A multiple regression framework was then utilized to understand the direct effects of school motivation and learning strategies on hazardous drinking behavior. Based on the work of Zeigler and colleagues^11^ examining underage drinking and study skills, it is hypothesized that hazardous drinking will be associated with greater study skill deficits and problematic learning strategies. The present study examines the following seven factors known to affect academic performance: study strategies, note-taking/listening skills, reading/comprehension strategies, writing/research skills, test-taking strategies, organization techniques, and time management. Based on extant research,^11^ we expect to document negative associations between hazardous drinking behavior and employing learning and study strategies effectively. In addition, relationships between hazardous drinking behavior and the following three student liabilities were explored: academic motivation, test anxiety, and concentration/attention. We expect to document positive associations between hazardous drinking behavior and the 3 student liabilities. The aim of this work is to identify potential treatment targets for individuals who report problematic hazardous drinking profiles.

## Method

### Participants

The sample consisted of 450 students (at least 18 years of age) enrolled in a psychology department research pool at a public, southeastern university. Participants were recruited by a clinical research laboratory through the SONA Research Participant Recruitment System, a cloud-based research participant pool. Students recruited through the SONA system received research participation credits for their course grade.

### Measures

Participants completed a demographic questionnaire to assess basic demographic information including age, handedness, gender, sex, race, educational history, health (physical and psychological) history, family history, and social history.

#### School Motivation and Learning Strategies Inventory College Form (SMALSI)

The SMALSI is a comprehensive self-report measure of learning skills and strategies.^14^ The SMALSI utilizes a 4-point Likert-type scale that ranges from 1 (“never”) to 4 (“always”) and assesses seven student strengths: study strategies (15 items), note-taking/listening skills (17 items), reading/comprehension strategies (12 items), writing/research skills (9 items), test-taking strategies (13 items), organizational techniques (13 items), and time management (14 items). In addition, the measure assesses 3 student liabilities: low academic motivation (26 items), test anxiety (25 items), and concentration/attention difficulties (20 items). The SMALSI has adequate internal consistency. The following Cronbach alphas are associated with SMALSI subscales: study strategies (α = .81), note-taking/listening skills (α = .81), reading/comprehension strategies (α = .79), writing/research skills (α = .73), test-taking strategies (α = .78), organizational techniques (α = .77), time management (α = .80), and time management (α = .80), low academic motivation (α = .91), test anxiety (α = .92), and concentration/attention difficulties (α = .84). Lower Student Strength scores indicate greater impairment. Lower Student Liabilities scores denote less difficulty.

#### Alcohol Use Disorders Identification Test (AUDIT)

The AUDIT is a 10-item assessment of hazardous drinking behavior.^19^ Participants were asked to provide information regarding alcohol-related consequences (eg, “Have you or someone else been injured because of your drinking?”), the frequency of alcohol consumption (eg, “How often do you have a drink containing alcohol?”), and the quality of alcohol consumption (eg, “How many drinks containing alcohol do you have on a typical day when you are drinking?”). The AUDIT has adequate internal consistency reliability (α = .76-.83)^20,21^ and good 1-week test–retest reliability (r = .84).^21^ In the present study, hazardous drinking behavior was defined as having an AUDIT score of 7 for men and 6 for women. Higher scores on the AUDIT denote greater hazardous drinking behavior.

### Procedure

IRB approval was obtained prior to data collection. On the university’s SONA Research Participant Recruitment website, participants were asked to view the study intent, general procedures, time involvement, potential benefits of participation, potential risks of participating, limits of confidentiality, and the right to cease participating in the study at any time without penalty or consequence. Students were invited to complete online questionnaires after consenting to study participation. Participants were invited to complete a lab-based research appoint after completing the online measures.

## Results

### Preliminary analyses

The mean age of participants was 18.59 years (*SD* = 0.81). With respect to gender, 268 participants (59.6%) identified as female, 179 participants (39.8%) identified as male, 2 participants (0.4%) self-identified as inter-gender, and 1 participant (0.2%) chose not to provide information regarding gender. Approximately 82.4% (n = 371) identified as white/Caucasian, 8.9% (n = 40) identified as black/African American, 5.6% (n = 25) identified as Asian, 1.1% (n = 5) identified as Native Hawaiian or Pacific Islander, and 2% (n = 9) of the sample chose not to provide information regarding race/ethnicity status. Of the enrolled participants, 75.3% (n = 339) identified as freshmen, 17.1% (n = 77) identified as sophomores, 5.1% (n = 23) identified as juniors, and 2.4% (n = 11) identified as seniors.

### Tier 1: bivariate correlations among study variables

Means, standard deviations, possible ranges of scores, and inter-correlations among study variables are presented in Table 1. Based on adopted AUDIT cutoff values,^22^ approximately 35.3% of the sample reported AUDIT scores in the clinical range. Bivariate relationships among study variables were examined to understand the relationships among study variables. Neither age (*P* = .05) nor gender (*P* = .10) correlated with hazardous drinking behavior. Hazardous drinking behavior was not related to class standing (*P* = .11). Hazardous drinking behavior correlated with the following 4 learning strategies: Note-taking/Listening Skills (r = –.159, *P* = .001), Test-taking Strategies (r = –.114, *P* *=* .015), Organization (r = –.163, *P* = –.001) and Time management (r = –.097, *P* *=* .039), suggesting that as hazardous drinking behavior increases, note-taking/listening, test-taking, organization, and time management abilities decrease. Hazardous drinking behavior was not related significantly to Study Strategies (*P* = .06), Reading/Comprehension Strategies (*P* = .07), or Writing/Research Skills (*P* = .48). Regarding student liabilities, hazardous drinking behavior correlated with both low academic motivation (r = .140, *P* = .003) and concentration/attention difficulties (r = .218, *P* < .001), suggesting that as hazardous drinking behavior increases, academic motivation and concentration/attention problems increase. Hazardous drinking behavior was not associated with test anxiety (*P* = .162) in the present study.

**Table 1.** Bivariate correlations among study variables.

		1	2	3	4	5	6	7	8	9	10	11	12
1	Age												
2	AUDIT	0.076											
3	Study	0.006	−0.089										
4	Note	−.105*	−.159**	.812**									
5	Read	−0.049	−0.085	.697**	.736**								
6	Write	0.033	−0.051	.665**	.564**	.591**							
7	Test	−0.04	−.114*	.829**	.753**	.649**	.640**						
8	Org	−0.076	−.163**	.753**	.716**	.545**	.496**	.676**					
9	Time	−0.05	−.097*	.788**	.728**	.628**	.552**	.707**	.793**				
10	Lomot	0.034	.140**	−.275**	−.250**	−0.086	−.192**	−.279**	−.257**	−.317**			
11	Tanx	−.104*	0.066	0.026	0.066	.096*	−0.053	0.045	−0.013	−0.089	.573**		
12	Condif	0.002	.218**	−.152**	−.249**	−.097*	−0.031	−.129**	−.297**	−.304**	.635**	.543**	
Mean		18.59	4.52	27.43	28.11	16.25	16.30	25.22	21.85	23.16	17.35	28.23	22.67
SD		0.81	5.14	8.29	8.96	6.28	5.29	7.41	7.18	7.65	11.64	13.18	9.99
Range		18-21	0-35	0-46	0-50	0-37	0-30	0-40	0-39	0-42	0-67	0-69	0-53

Higher scores on the AUDIT denote greater hazardous drinking behavior. Lower Student Strength scores indicate greater impairment. Lower Student Liabilities scores denote less difficulty. Abbreviation: AUDIT, Alcohol Use Disorders Identification Test; Condif = Concentration/Attention Difficulties; Lomot = Low Academic Motivation; Note = Note-Taking/Listening Skills; Org = Organizational Techniques; Read = Reading/Comprehension Strategies, Study = Study Strategies; Tanx = Test Anxiety; Test = Test-taking Strategies, Time = Time Management; and Write = Writing/Research Skills.

* *P* < .05. ** *P* < .01. ***P < .001

### Tier 2: multiple linear regressions

Multiple linear regressions were conducted to examine the direct effects of both learning strategies (adjusted R^2^ = 0.024) and student liabilities (adjusted R^2^ = 0.045) on hazardous drinking behavior. Regarding student strengths, organizational techniques (partial correlation coefficient = –.163) was the only significant predictor of hazardous drinking behavior after controlling for both gender and age, *b* = –.163, *t*(448) = 12.23, *P* = .001. Study strategies, note-taking/listening skills, reading/comprehension strategies, writing/research skills, test-taking strategies, and time management were not significant predictors of hazardous drinking behavior. Regarding student liabilities, only concentration/attention difficulties (partial correlation coefficient = .218) predicted AUDIT scores significantly after controlling for both gender and age, *b* *=* .112, *t*(448) = 22.36, *P* < .001 (see Table 2). Neither low academic nor test anxiety predicted hazardous drinking behavior.

**Table 2.** Regression analysis summary: Alcohol Use Disorders Identification Test (AUDIT).

Model	Variable	AUDIT			β	*t*	*p*
		R	R^2^	SE			
1.^a^		.163	.027	5.08			
	(Constant)				7.07	9.21	<.001
	Study				.08	1.09	.277
	Note				−.09	−1.30	.196
	Read				.01	.10	.923
	Write				.04	.74	.460
	Test				−.01	−.12	.902
	Organize				−.16	−3.50	.001
	Time				.09	1.13	.259
1.^b^		.218	.048	5.03			
	(Constant)				1.98	3.36	.001
	Lomot				.003	.051	.959
	Tanx				−.074	−1.35	.176
	Condif				.112	4.73	<.001

a Predictors: (Constant), Study strategies (study); Note-taking/listening skills (note); Reading/Comprehension strategies (read) Writing/research skills (write); Test-taking strategies (test); Organizational techniques (organize); and Time management (time).

b Predictors (Constant), Low Academic Motivation (lomot); Test Anxiety (tanx); and Concentration/Attention Difficulties (condif).

## Discussion

The present investigation aims to understand the associations among hazardous drinking behavior, academic skills/strategies, and student liabilities. The SMALSI was utilized to assess 7 learning skills/strategies: study strategies, note-taking listening skills, reading/comprehension strategies, writing/research skills, test-taking strategies, organizational techniques, and time management.^14^ Understanding academic skills/strategies is important, as learning skills/strategies are linked to both academic achievement and improved overall functioning for college students.^14^ Moreover, 3 student liabilities were assessed in the present study: low academic motivation, test anxiety, and concentration/attention difficulties. An understanding of student liabilities (ie, academic motivation, test anxiety, and concentration/attention difficulties) will inform the identification for potential treatment targets for populations at risk of engaging in hazardous drinking behavior. To our knowledge, this is the first examination, to date, to examine the associations among hazardous drinking behavior, academic skills/strategies, and student liabilities.

Based on the adopted AUDIT criteria,^22^ more than a third of participants in our sample exhibited AUDIT scores in the clinical range (35.3%). It is important to note that most of the study participants were freshmen. Targeted recruitment efforts were not adopted by the research team (ie, current drinkers were not recruited) in this pilot investigation. Traditionally, freshmen fall between 17 and 20 years of age.^23^ This group is below the legal drinking age and rates/frequency of hazardous drinking behavior may change as this population transitions to upperclassmen status. Hansson and colleagues^24^ found that approximately 54% of college students (average age 25 years) have scores above the AUDIT cutoff. These participants reported having at least 1 parent with self-reported alcohol problems.^24^ In a sample of 401 current drinkers, DeMartini and Carey^22^ found that 52% of the sample met criteria for the at-risk status. Our findings suggest that it is important for campus-based interventions to be mindful of this high-risk/vulnerable population, as treatment gains obtained during the freshmen year may inform future academic success and improve the academic outlook. Research suggests that alcohol consumption during the freshman year is predictive of academic problems throughout the college years.^25–27^ Future research should examine the extent to which Greek membership and/or common concerns experienced by college students (eg, anxiety and depression) may impact college drinking behavior.

Moreover, consistent with research linking substance use/abuse to impaired learning,^11^ our findings suggest that hazardous drinking behavior is associated with 4 of 7 learning strategies. Our findings are consistent with research suggesting a link between hazardous drinking behavior and both learning and memory.^11^ It is important for future research to examine whether higher-order cognitive processes (ie, executive functions) moderate the relation between learning strategies and hazardous drinking behavior. Note-taking is a “developmental process” that encompasses encoding/storing information and monitoring comprehension with self-questioning.^28^ Evidence-based methods used to improve note-taking skills include using shorthand writing methods to train individuals to write faster, reviewing subject material before class, writing questions about the study material, and using guided lecture notes provided by the professor.^29^ Test-taking strategies, however, aim to improve performance by familiarizing students with the testing situation and reviewing test formats.^30^ At least 6 evidence-based test-taking strategies have been identified: using retrieval cues, adopting time strategies, avoiding common errors, appropriate guessing techniques, deductive reasoning skills, and considering the intent of the question.^31^ Time management, however, involves effectively managing time to complete tasks/assignments and estimating the time needed to complete goals and related subtasks. Researchers posit that time management is the “self-management of behavior” and individuals must be mindful of schedules, goals, and required tasks for goal completion at all times. Time management should also include dividing complex tasks into management subtasks and designated incentives/rewards for task completion.^32^ The significant association between hazardous drinking behavior and time management in the present study is consistent with research on hazardous drinking behavior and completing assignments in a timely fashion, missing class, and being late for class.^7^ In the present study, only organization techniques predicted hazardous drinking behavior after controlling for both gender and age. Organization/planning is a higher-order cognitive process that is linked to both attention problems and hazardous drinking behavior. Researchers posit that organization involves 3 components: time, object, and idea.^33^ Adequate time should be devoted to identify the required materials and brainstorm. Organization includes the following behaviors: (a) managing tasks within a specified time context, (b) arranging supplies for assignments within space for easy access and deployment, and (c) configuring a systematic approach to a task.^33^

In the present study, hazardous drinking behavior was not related to study strategies, reading/comprehension strategies, or writing/research skills. While research links substance use to retrieval problems for both verbal and visual material,^18^ we did not document an association between hazardous drinking behavior and reading/comprehension strategies. It is important to note that Brown and Tapert^18^ examined the attention skills and the retrieval of both verbal and nonverbal material in a sample of adolescents with alcohol use disorders. We did not document an association in our older, nonclinical sample.

Regarding student liabilities, hazardous drinking behavior was associated with both low academic motivation and concentration/attention difficulties in the present study. An association between hazardous drinking behavior and test anxiety was not documented in the present study. According to researchers,^34^ academic motivation requires skill, self-regulation, willpower (ie, motivation to achieve), and investment in the process of learning. Motivated students are “empowered learners”^35^ who expect to complete tasks/goals and esteem the learning experience. Self-efficacy^36^ and student attributions^37,38^ are central to evidence-based strategies to improve academic motivation. Moreover, attention/concentration abilities are central to learning, as attention is a precursor to encoding, processing, and retrieving information.^39^ Attention is a multifaceted construct that includes divided attention, sustained attention, and focused attention.^40^ Evidence-based strategies aimed at improving attention/concentration abilities target identifying important/relevant information, ignoring irrelevant information, allocating attention, and monitoring understanding/comprehension.^41^ The unique association between attention/concentration abilities and hazardous drinking behavior is consistent with research on substance use and concentration in the classroom environment.^7^ Individuals with chronic and debilitating attention problems (ie, individuals diagnosed with attention deficit hyperactivity disorder [ADHD]) are more likely to use substances,^42^ with both attention problems and impulsivity being related to alcohol use/abuse.^43^ Campus-based intervention for individuals diagnosed with ADHD should consider comorbid mental health concerns (ie, substance use) and underlying learning challenges unique to this population. In the present study, concentration/attention difficulties were related to hazardous drinking behavior and 7 learning strategies: study strategies, note-taking/listening skills, reading/comprehension strategies, test-taking strategies, organizational techniques, and time management. Similarly, concentration/attention difficulties were related to both low academic motivation and text anxiety. It is important for future research to assess and treat (if necessary) comorbid hazardous drinking behavior in college students with chronic attention problems that impair functioning.

While our findings suggest that note-taking/listening skills, test-taking strategies, organizational techniques, time management, academic motivation, and concentration/attention abilities are potential treatment/interventions for individuals at risk of engaging in hazardous drinking behavior, several methodological concerns should be considered. First, this cross-sectional design relied exclusively on self-report data. It is important for future research to examine longitudinally the development of learning strategies and student liabilities across the college experience, with special consideration of higher-order cognitive processes (ie, executive functions) that underlie/support learning strategies and student liabilities. In addition, most of the participants in the present study identified as freshmen (75.3%), as participants were largely recruited from lower-level classes that require research participation. Finally, it is important for future work to consider student academic records and objective measures both hazardous drinking behavior and learning strategies in a more diverse sample. Students at risk of engaging in hazardous drinking behavior should be evaluated in a lab and/or applied setting (eg, classroom) to understand the associations among attention problems, learning strategies, and hazardous drinking behavior. In addition, the utilization of external collateral information from multiple informants (eg, teachers/instructors, roommates, parents, significant others) and controlling for positive impression management would lend support to our finding that attention problems predict hazardous drinking behavior.
